# The Effect of Resveratrol on Surgery-Induced Epidural Fibrosis in Laminectomy Rats

**DOI:** 10.1155/2014/574236

**Published:** 2014-03-24

**Authors:** Peifeng Sun, Bo Miao, Hongmei Xin, Jinzhu Zhao, Guofeng Xia, Peng Xu, Jian Hu, Zhenfeng Li, Jianmin Li

**Affiliations:** ^1^Shandong University, Jinan 250100, China; ^2^Department of Orthopedics, Chinese People's Liberation Army 401 Hospital, Tsingtao, Shandong 266071, China; ^3^Department of Orthopedic Trauma & Hand and Foot Surgery, Jinan Central Hospital Affiliated to Shandong University, Jinan 250100, China; ^4^Qilu Hospital of Shandong University, Tsingtao 266010, China; ^5^Qilu Hospital of Shandong University, Jinan 250012, China

## Abstract

Epidural fibrosis (EF) is a common complication for the patients who underwent laminectomy. Recently, EF is thought to cause recurrent postoperative pain after laminectomy. Resveratrol has been shown to exert its anti-inflammatory, antifibrotic, and antiproliferative multifaceted properties. The object of this study was to investigate the effects of resveratrol on the prevention of postlaminectomy EF formation in laminectomy rats. A controlled double-blinded study was performed on 60 healthy adult Sprague-Dawley rats that underwent lumbar laminectomy at the L1-L2 levels. They were divided randomly into 3 groups (1, 2, and 3) of 20 rats each—group 1: resveratrol treatment group; group 2: resveratrol dilution saline treatment group; group 3: sham group (rats underwent laminectomy without treatment). All rats were killed 4 weeks after operation. The Rydell score, hydroxyproline content, vimentin cells density, fibroblasts density, and inflammatory factors expressional levels all suggested better results in resveratrol group than the other two groups. Resveratrol is able to inhibit fibroblasts proliferation, and TGF-**β**1 and IL-6 expressions and prevent epidural fibrosis in postlaminectomy rat.

## 1. Introduction

Over one million patients all over the world undergo lumbar laminectomy for treating disc herniation, making it one of the most widely accepted treatments for lumbosacral disorders [1, 2]. Subsequently, failed back surgery syndrome (FBSS) was reported. With the characteristics that patients could suffer from the recurrence of continued pain in the lower posterior trunk and/or lower extremities after lumbar laminectomy, FBSS could lead to the failure of the operation [3]. As early as in 1948, EF was first mentioned to be a scar tissue adjacent to the dura mater following lumbar laminectomy [4]. In 1983, some researchers pointed out that EF might be a contributing factor for FBSS [5].

Until the present, an established EF is still a great challenge to surgeons, and reoperations seem to be helpless and risky [6]. Currently, extensive studies have been tried out on animals with several differing components, such as Adcon-L, free fat grafts, gelfoam, pharmaceutical gels, anti-inflammatory agents, anticancer agents, honey, and others [7–12]. Although some of them have achieved a certain level of success in animals, there is still no optimal solution which has gained success in clinical application or wide acceptance.

Resveratrol (3,5,4′-o-trihydroxystilbene), which is found in grapes, red wine, various other fruits, and medicinal plants, has exerted its variety of biological properties including anticarcinogenic, antioxidant, and anti-inflammatory in a number of previous literatures [13–15]. Also, it can cause vasodilatation and inhibit platelet aggregation [16, 17]. A group of studies have reported that resveratrol is able to prevent the postoperative scar adhesions in animals [16, 18, 19]. After an extensive review, this is the first study on the effect of resveratrol on prevention EF.

In our laminectomy rat model, we investigated whether resveratrol attenuates EF by regulating the expressions of inflammatory factors, such as interleukin 6 (IL6), transforming growth factor-*β*1 (TGF-*β*1), and hydroxyproline, which are suggested to be involved in the promotion of EF.

## 2. Material and Methods

### 2.1. Animals

60 healthy male Sprague-Dawley rats (mean weight = 280 g) were employed for this study. Experiments were carried out in compliance with the principles of International Laboratory Animal Care and with the European Communities Council Directive (86/809/EEC) and were approved by the local ethical committee. All rats were randomly divided into three groups (twenty rats per group): (1) resveratrol treatment group; (2) saline treatment group; (3) sham group (laminectomy without treatment).

### 2.2. Reagents and Antibodies

Both resveratrol and *β*-dimethylaminobenzaldehyde were purchased from Sigma Aldrich Corp., St. Louis, MO, USA. Cal-EX II solution for decalcification and dehydration was purchased from Thermo Fisher Scientific (Waltham, MA, USA), Orangeburg. Reverse transcriptase was purchased from Promega (Madison, WI, USA). Primary anti-vimentin antibody (ab92547) was purchased from abcam. Secondary antibodies were purchased from Santa Cruz.

### 2.3. Surgery

Lumbar laminectomy was grimly performed according to previous protocol under sterile conditions employing basic surgical tools [20]. Basically, all rats were numbered, respectively, and anesthetized by intraperitoneal injection of 10% chloral hydrate (0.3 mL/100 g body weight). The hairs around the L1 and L2 were shaved and the exposed skin was sterilized. A median incision of dorsal skin was made and the paraspinal muscles were separated on L1-L2 vertebrae. The dura mater of L1 vertebrae was exposed after removing the spinous process and vertebral plate with a rongeur. Gauze was used for homeostasis. The wound site was surgically closed. Close attention was paid not to injure the dura and the nerve roots. Resveratrol (6 mg/kg/day) for group 1 or saline for group 2 was given by the orogastric route to the laminectomy rats for 20 days [18].

### 2.4. Macroscopic Assessment

Macroscopic assessment was performed 4 weeks after surgery. Five rats were randomly selected from each group and anesthetized. With the help of assistants, the epidural scar adhesion underwent double-blind evaluation. And the results were classified based on the Rydell classification (Table 1) [21].

### 2.5. Evaluation of Hydroxyproline Content (HPC) in Epidural Scar Tissue

Hydroxyproline content (HPC) analysis was performed 4 weeks after surgery. Five rats were randomly selected from each group and anesthetized. The scar tissue approximately 5 mg wet weight was obtained around the wound site. The content of hydroxyproline of different groups in scar tissue was examined according to the previous protocol [22]. Basically, the samples were lyophilized, ground, and hydrolyzed with 6 mol/L HCl at 110°C for 24 hours. Then 1 mL hydroxyproline developer (*β*-dimethylaminobenzaldehyde solution) was added to the samples and the standards. The absorbances at 550 nm were read using a spectrophotometer. Finally, the hydroxyproline content (HPC) per milligram of scar tissue was calculated according to the standard curve constructed by the serial concentration of commercial hydroxyproline.

### 2.6. Histological Analysis

Histological analysis was performed 4 weeks postoperatively. Five rats were randomly selected from each group and anesthetized. Intracardial perfusion with 4% paraformaldehyde and saline was performed. The whole L1 vertebral column including the paraspinal muscles and epidural scar tissue was resected and fixed in 10% phosphate-buffered formaldehyde solution. Cal-Ex II solution was employed to decalcify and dehydrate the samples. After that, they were embedded in paraffin, and 5 *μ*m axial sections of the wound site were stained with hematoxylin and eosin (H&E). The epidural scar adhesion was evaluated under the light microscope (Leica CM3050S, Germany). Three counting areas were selected at the middle and at the margins of the laminectomy sites. The number of fibroblasts cells was calculated. To be more stringent, in order to further quantify the fibroblasts numbers, the immunohistochemistry was performed with application of the monoclonal anti-vimentin antibody and the density of vimentin was evaluated.

### 2.7. Analysis of IL-6 and TGF-*β*1 Concentrations

The mRNA analyses of IL-6 and TGF-*β*1 were performed four weeks postoperatively. Five rats randomly selected from each group were killed, and the scar tissues from the laminectomy sites were collected. Total RNA was extracted using TRIzol reagent and the RNA (2 *μ*g) was transcribed into cDNA by use of AMV Reverse Transcriptase. Quantitative real-time PCR (RTPCR) was performed according to previous study using the Bio-rad MYIQ2 (USA) [23]. Primer sequences were TGF-*β*1 (148 bp): forward, 5′-GCCCTGCCCCTACATTTGG-3′; reverse, 5′-CTTGCGACCCACGTAGTAGACGAT-3′; IL-6 (131 bp): forward, 5′-ACCCCAACTTCCAATGCTCT-3′; reverse, 5′-TGCCGAGTAGACCTCATAGTGACC-3′; GAPDH (169 bp): forward, 5′-TCACCACCATGGAGAAGGC-3′; reverse, 5′-GCTAAGCAGTTGGTGGTGCA-3′. GAPDH amplification was used as an internal control.

### 2.8. Statistical Analysis

The statistical analysis was performed using SPSS 13.0 statistical package (SPSS Inc., Chicago, IL, USA). Data are expressed as mean ± standard deviation values. The single factor analysis of variance (ANOVA) and *q*-test were applied to evaluate three independent samples. Statistical significance was assumed at *P* < 0.05.

## 3. Results

### 3.1. Macroscopic Assessment of Epidural Scar Adhesion

The laminectomy was well tolerated by all rats without wound infection, neurological deficit, and cerebrospinal leak.

In the resveratrol group, macroscopic observation showed that soft or weak fibrous adhesion was observed in the laminectomy sites. However, in the laminectomy sites of rats treated with saline or nothing, severe epidural scar adhesions were seen. And it was difficult to dissect the scar adhesions accompanied by bleeding and disruption of the dura mater. The grades of epidural scar adhesion in rats were evaluated according to the Rydell classification (Table 2).

### 3.2. Hydroxyproline Content (HPC)

**Figure 1:**
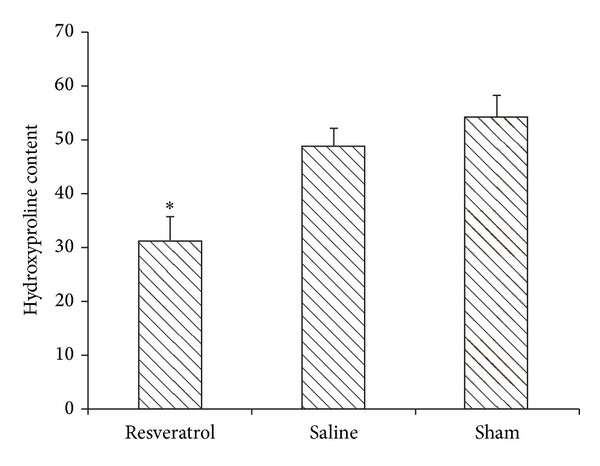
Hydroxyproline levels were expressed as the mean ± standard deviation of hygrotissue. The resveratrol group showed a least hydroxyproline level. *P < 0.01, compared with other two groups.

HPC concentration in epidural scar tissue was shown in Figure 1. The resveratrol group (31.32 ± 4.72 *μ*g/mg) showed a significant reduction compared with that of the saline group (48.92 ± 3.54 *μ*g/mg, *P* < 0.01) and sham group (54.17 ± 4.39 *μ*g/mg, *P* < 0.01). The content in saline group showed no significant difference compared with that of sham group (*P* > 0.05).

### 3.3. Histological Analysis

**Figure 2:**
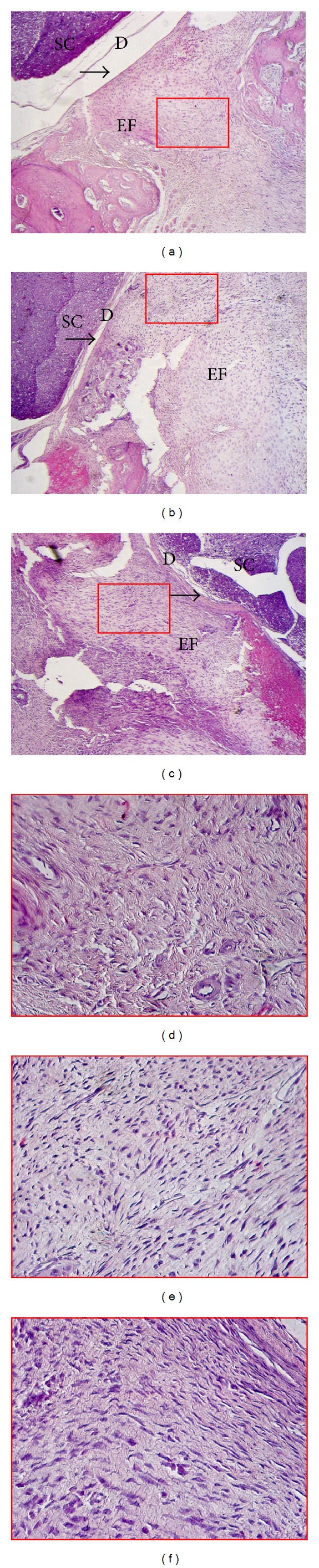
H&E staining for the epidural scar tissues in the laminectomy sites employed with resveratrol (a), saline (b), and the sham groups (c). (a) Loose scar tissues without adherence (as right arrow pointed) to dura mater were observed in the resveratrol group. (b, c) Dense scar tissues (as right arrow pointed) adhered to dura maters were observed in both saline and sham groups. (d, e, and f) The fibroblasts in the epidural fibrosis were seen by further magnification. The magnification of (a), (b), and (c) was 100x; the magnification of (d), (e), and (f) was 400x. SC: spinal cord, D: dura mater, and EF: epidural fibrosis.

In the laminectomy sites of the saline group and sham group, dense epidural scar tissue with widespread adhesions to dura mater and dorsal muscle was observed (Figures 2(b) and 2(c)). However, in the laminectomy sites of the resveratrol group, loose or little scar adhesion was seen (Figure 2(a)).

The number of fibroblasts in the resveratrol group (122.98 ± 25.44) was significantly less than those of the saline group (278.39 ± 31.27) and sham group (292.16 ± 30.65). Representative sections are shown in Figures 2(d), 2(e), and 2(f).

### 3.4. Effect of Resveratrol on Vimentin

**Figure 3:**
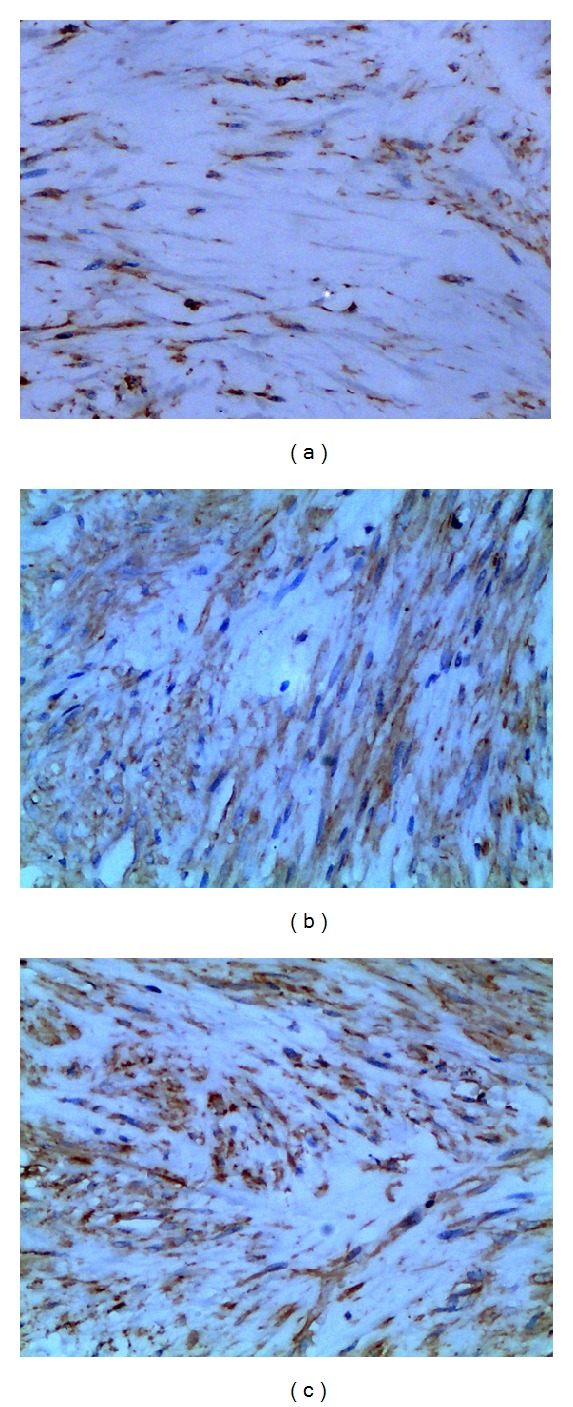
Immunohistochemistry analysis of vimentin cells in epidural scar tissues employed with resveratrol (a), saline (b), and the sham groups (c). Less positive vimentin in the resveratrol group (a) was less than those of the other 2 groups. The density of positive vimentin in the saline group was similar to that of sham group. The magnification was 400x.

In an effort to be more definitive, an additional immunohistochemistry analysis for vimentin was conducted. Less positive vimentin was observable in the resveratrol group than the saline and sham group (Figure 3).

### 3.5. Effect of Resveratrolon IL-6 and TGF-*β*1

**Figure 4:**
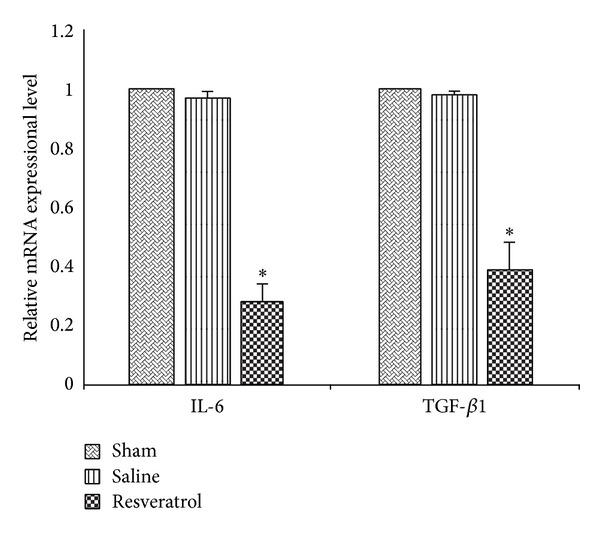
The mRNA expressional level of IL-6 and TGF-*β*1 in epidural scar tissue in each group. RT-PCR testing was conducted to evaluate the relative mRNA expressional level. *P < 0.01, compared with sham group.

In order to find out the effect of resveratrol's anti-inflammatory activity in EF rat, RTPCR on examining the mRNA expressional levels of both TGF-*β*1 and IL-6 was conducted. The results of mRNA expressional levels of both TGF-*β*1 and IL-6 are shown in Figure 4: the resveratrol group was lower than those of the saline group (*P* < 0.01) and sham group (*P* < 0.01); the expressions between saline group and sham group were not significantly different (*P* > 0.05).

## 4. Discussion

Historically, the literature reports that the incidence rate of FBSS among lumbar discectomy patients was 5–30% [24]. Many factors can lead to FBSS, such as improper diagnosis and surgery, disc herniation, spinal stenosis, recurrent or retained disc, and epidural fibrosis [5, 25, 26]. Epidural fibrosis, as an epidural scar tissue, is characterized by extracellular matrix protein deposition, fibroblast accumulation, and distortion of normal tissue architecture due to inflammation. It can cause extensive adhesions around the dura mater and squeeze the nerve roots [23, 27]. Besides, it is a risk factor for surgeons to perform repeat operations, as it may lead to dura tearing, excessive bleeding, and nerve root injury [12]. Therefore, it is important to reduce the three aforementioned factors in order to effectively prevent EF.

In the present study using sixty EF rats, resveratrol has demonstrated its high efficacy in multifaceted suppression of the factors. The data showed significant decrease of hydroxyproline levels in scar tissues. The Rydell score showed a better result treated with resveratrol than the other 2 groups. Both H&E staining and further immunohistochemistry analysis for vimentin enhanced our hypothesis. RTPCR analyses showed a significant decrease of both IL-6 and TGF-*β*1. All of the above support resveratrol's antifibrotic, anti-inflammatory, and antiproliferative roles. Literatures also support our theory by reporting on resveratrol's antifibrotic, anti-inflammatory, and antiproliferative properties in other surgery-induced adhesions [15, 16, 18]. These studies and present data may explain some if not all of the possible mechanisms that make resveratrol effective in preventing or suppressing EF.

After further extensive literature reviews, we feel confident to report that the present study may be one of the first studies studying resveratrol's suppressive effects on EF by down regulating inflammatory expressional levels such as IL-6 and TGF-*β*1 and also reducing hydroxyproline deposition in rats. Undoubtedly, in further research, more research on drug safety, effectively safe concentration, long-term effects, and possible side and adverse effects of resveratrol is needed before clinical trials and application.

## Figures and Tables

**Table 1:** Rydell classification.

Grade 0	Epidural scar tissue was not adherent to the dura mater
Grade 1	Epidural scar tissue was adherent to the dura mater but easily dissected
Grade 2	Epidural scar tissue was adherent to the dura mater and difficultly dissected without disrupting the dura matter
Grade 3	Epidural scar tissue was firmly adherent to the dura mater and could not be dissected

**Table 2:** Grades of epidural adhesion in rats, according to the Rydell classification.

Group	Grade			
	0	1	2	3
Resveratrol	4	1	0	0
Saline	0	0	2	3
Sham	0	0	0	5
